# Syphilitic Re-infection of Husband and Wife

**Published:** 1883-06

**Authors:** 


### Syphilitic Re-infection of Husband and Wife.

The London *Medical Record* says that Dr. C. Pellizzari, of Florence, reports the following case (*Lo Sperimentale*):

About the middle of December, 1880, a healthy-looking married man, about fifty years of age, consulted Dr. Pellizzari for phimosis, with discharge from beneath the prepuce, and enlarged inguinal glands, which had followed a suspicious intercouse some days before. Induration of the corona subsequently became well-marked, and in due course a macular syphilide appeared, having been preceded by osteocopic pains. The man, on being told that he was suffering from syphilis, remarked that he had suffered from venereal sores ten years before, and had also at that time infected his wife, who, a few months afterwards, during pregnacy, had suffered from general debility, headache, and moist papules of the genital organs. Her child also showed signs of syphilis soon after its birth. Further and positive evidence of the wife's infection was obtained from Professor P. Pellizzari, who had attended her in 1873 for syphilitic perforation of the septum nasi. On February 2, 1881, she was examined by the author, and found to have an indurated sore of the fourchette and enlarged inguinal glands; a month later a maculo-papular syphilide appeared. The two attacks of syphilis in the case of the wife are thus clearly proved; but as regards the husband, the evidence of the former attack appears to rest on the fact stated by the man himself, that he had ten years previously contracted venereal sores after suspicious intercourse, and on the proof of subsequent syphilis in the wife and child. The husband appears to have suffered so slightly on that occasion, that he did not think it necessary to obtain medical advice. In connection with this it may be mentioned that, in the later attack, when he was under the author's observation, the general symptoms were mild in degree; while the wife suffered severely from recurrent eruptions, nodes, etc., although she had been almost continuously under treatment during the preceding ten years.—*Med. & Surg. Reporter.*

